# Family Planning Decision Making in People With Multiple Sclerosis

**DOI:** 10.3389/fneur.2021.620772

**Published:** 2021-04-28

**Authors:** Simona Bonavita, Luigi Lavorgna, Hilary Worton, Susan Russell, Dominic Jack

**Affiliations:** ^1^Department of Advanced Medical and Surgical Sciences, University of Campania “Luigi Vanvitelli,” Naples, Italy; ^2^Neurological Institute for Diagnosis and Care “Hermitage Capodimonte,” Naples, Italy; ^3^Aequus Research, London, United Kingdom; ^4^EMD Serono Research & Development Institute, Inc., Billerica, MA, United States; ^5^Merck KGaA, Darmstadt, Germany

**Keywords:** multiple sclerosis, family planning, pregnancy, survey, disease-modifying drugs

## Abstract

**Introduction:** The majority of people diagnosed with MS are of childbearing or child fathering age, therefore family planning is an important issue for both women and men with MS. Fertility and the course of pregnancy are not affected by MS; however, people with MS (pwMS) may have concerns that there will be a greater risk of complications to the mother and/or adverse pregnancy outcomes either due to the disease or to ongoing medication. This survey aimed to understand family planning decision making in pwMS and related unmet educational needs.

**Methods:** A total of 332 pwMS across the USA, UK, France, Germany, Italy, and Spain were recruited from a specialist patient panel agency to participate in a smartphone-enabled standing panel. The 80-question survey focussed on decision making and information sources for pwMS regarding family planning, as well as behavior during and after pregnancy. Male patients with MS did not respond to specific questions on pregnancy. Survey results were directly compared with the 2016 US and 2010 UN census data.

**Results:** pwMS were more likely to have no children than the general population, particularly in the subgroup of patients aged 36–45 years. A total of 56% of pwMS reported that the disease affected, with different degrees of impact, their family planning decision making. Of these, 21% significantly changed their plans for timing of pregnancy and the number of children, and 14% decided against having children. Participants indicated that healthcare professionals were the primary source of information on family planning (81% of responses). The timing of planned pregnancy was not considered when selecting treatment by 78% of participants.

**Conclusion:** MS was found to significantly impact family planning decision making, with pwMS significantly less likely to have children in comparison with the general population.

## Introduction

Multiple sclerosis (MS) is a chronic neurological illness, found to be more common in women than men. The majority of people diagnosed with the disease are of childbearing or child fathering age; therefore family planning is an important issue for both women and men with MS (1). Fertility and the course of pregnancy are generally not affected by MS (2); however, people with MS (pwMS) may have concerns that there will be a greater risk of complications to the mother and/or adverse pregnancy outcomes either due to the disease or to ongoing disease-modifying therapy (DMT).

The number of available DMTs has created a need for a risk-benefit analysis to be conducted between women with MS (wMS) who are considering pregnancy and their treating physician (3), as the risk-benefit profile varies between different DMTs. In wMS that are contemplating pregnancy, it is necessary to evaluate the impact that treatment cessation may have on the course of their disease. For the majority of DMTs, it is generally recommended that wMS stop treatment before becoming pregnant, and do not re-start until after they have stopped breastfeeding (4). However, this puts the patient at risk of recurrence or rebound of disease activity. A prolonged washout period before pregnancy increases the overall length of time that the patient is left unprotected against the risk of relapses (5). Recent studies have suggested that some DMTs do not require a washout period before attempting pregnancy (6) and case studies have shown that certain DMTs have been used during pregnancy for women with higher MS activity, with minimal negative reactions (7, 8). Furthermore, registry data have shown that exposure to DMTs in pregnant wMS caused minimal to no differences in birth weight, length, premature birth, or other adverse pregnancy outcomes compared with women with no DMT exposure (9–11). From the male perspective, formal reports on the effects of DMTs on semen quality are lacking (12), therefore, fertility issues may influence family planning decision making in men with MS.

Investigations into the long-term effects on disability have been carried out on pregnant wMS. Studies have shown that there is no evidence that parity influences the risk of secondary progression in MS (13). Pregnancy was also shown to not affect the time to reach an Expanded Disability Status Scale (EDSS) score of 6.0 (14), a marker of disability progression where assistance is required for the patient to walk up to 100 m (15).

Studies assessing how MS affects family planning decision making for pwMS are scattered. It has been suggested that several MS-related factors may influence family planning decision making, including the impact of MS on the patient's quality of life (16), or treatment options before and during pregnancy (17). However, the influence of such factors on family planning are rapidly changing with the increase in possible treatments, where patients are much less disabled than they were previously. This survey aimed to understand family planning decision making in pwMS and related unmet educational needs.

## Methods

### Patient Survey

A total of 332 pwMS across the USA (*n* = 76), UK (*n* = 51), France (*n* = 53), Germany (*n* = 50), Italy (*n* = 51), and Spain (*n* = 51) were recruited from a specialist patient panel agency to participate in a smartphone-enabled standing panel. The patients had previously signed up to join the research panel and were contacted by email to participate in this study. The survey was carried out over 6 months in 2018. Participants committed between 5 and 10 min approximately every 2 weeks to respond to survey questions. The response time was variable between patients. Responses were obtained from each participant before further surveys were released to the smartphone-enabled standing panel. Different topics were researched, focussing on real moments that matter throughout the patient's day/week and linked to individual patients, their medications, and symptoms.

The survey included 80 questions focussing on decision making and information sources for pwMS considering family planning, as well as behavior during and after pregnancy. Male patients did not respond to specific questions on pregnancy. MS phenotype and disability status were not collected as part of the survey. The survey format included multiple-choice questions, where several information sources could be selected. Respondents were asked to provide details on information sources used when considering family planning; these sources were graded based on how important they were when they were considering family planning. Importance was graded from “not at all important” to “very important” (Supplementary Material).

A research ethics committee review was not required, as the survey was conducted by professional market researchers and complied with relevant national guidelines for such research.

### Inclusion Criteria

To be eligible for study inclusion, participants were required to be aged between 18 and 65 years, diagnosed with MS for at least 2 years, and currently taking medication for MS. Older women, i.e., those in the 45–65 years' bracket, were included in the sample to ensure that the study had a retrospective view of their family planning decisions to compliment those of younger participants. Patients were also required to have no affiliation with pharmaceutical companies or not to have participated in similar research studies within the past 3 months before study entry. To be included in the analysis of study results, participants were required to have answered ≥ 80% of survey questions (18).

### Census Data

Survey results were directly compared with 2016 US census data and 2010 UN census data (19, 20). As this was a descriptive study, and census aggregate data contained no standard deviation, no statistical methods were involved during the comparison.

### Data Availability Statement

Any requests for data by qualified scientific and medical researchers for legitimate research purposes will be subject to Merck KGaA's Data Sharing Policy. All requests should be submitted in writing to Merck KGaA's data-sharing portal https://www.merckgroup.com/en/research/our-approach-to-research-and-development/healthcare/clinical-trials/commitment-responsible-data-sharing.html. When Merck KGaA has a co-research, co-development, or co-marketing or co-promotion agreement, or when the product has been out-licensed, the responsibility for disclosure might be dependent on the agreement between parties. Under these circumstances, Merck KGaA will endeavor to gain agreement to share data in response to requests.

## Results

### Participant Characteristics

**Figure 1:**
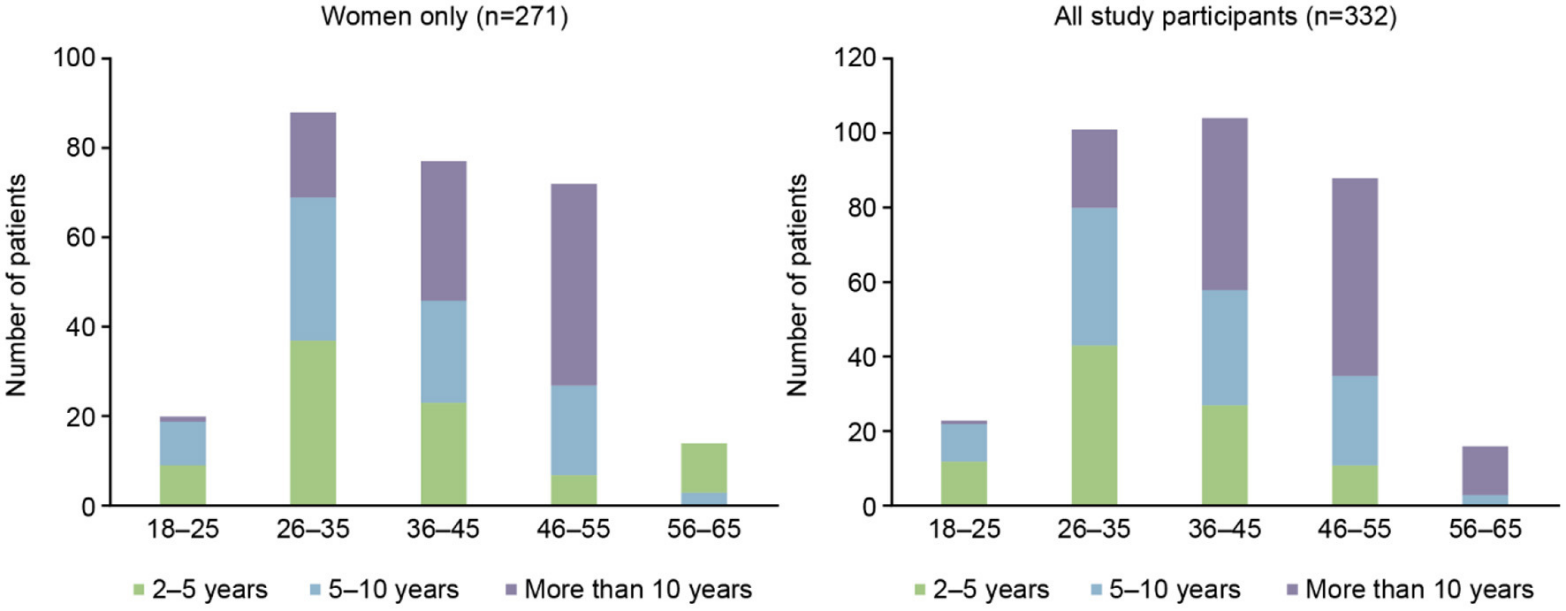
Time since diagnosis of multiple sclerosis, by age group.

Out of 332 participants, 271/332 (82%) were female and of these 185/271 (68%) were of childbearing age (18–45 years); 20/271 (7%) were in the 18–25 years of age subgroup, 88/271 (32%) were 26–35 years of age, and 77/271 (28%) were in the 36–45 years of age subgroup. The time since MS diagnosis by age group for the overall population and females only can be seen in Figure 1. The current medication of the participants at the time of the survey is shown in Table 1, with dimethyl fumarate, fingolimod, and natalizumab being the most commonly used DMTs. A larger number of patients had been taking their current medication for an extended period of time, with 76/332 (23%) taking their current medication for 5 or more years (Table 2).

**Table 1:** Current use of DMTs among survey participants (*N* = 332).

**DMT**	**Number of patients, *n***
Dimethyl fumarate	63
Fingolimod	62
Natalizumab	60
Glatiramer acetate	42
Alemtuzumab	22
Interferon β-1a	22
Ocrelizumab	21
Teriflunomide	21
Peginterferon β-1a	10
Interferon β-1b	7
Cladribine tablets	1
Mitoxantrone	1

*DMT, disease-modifying therapy*.

**Table 2:** Length of time on current DMT among survey participants (*N* = 332).

**Length of time since starting current DMT**	**Number of patients, *n***
Within the past month	10
1–3 months ago	16
4–6 months ago	18
6–12 months ago	25
13–24 months ago	43
2–3 years ago	64
3–5 years ago	70
More than 5 years ago	76
Unknown	10

*DMT, disease-modifying therapy*.

### Proportion of Participants With Children

**Figure 2:**
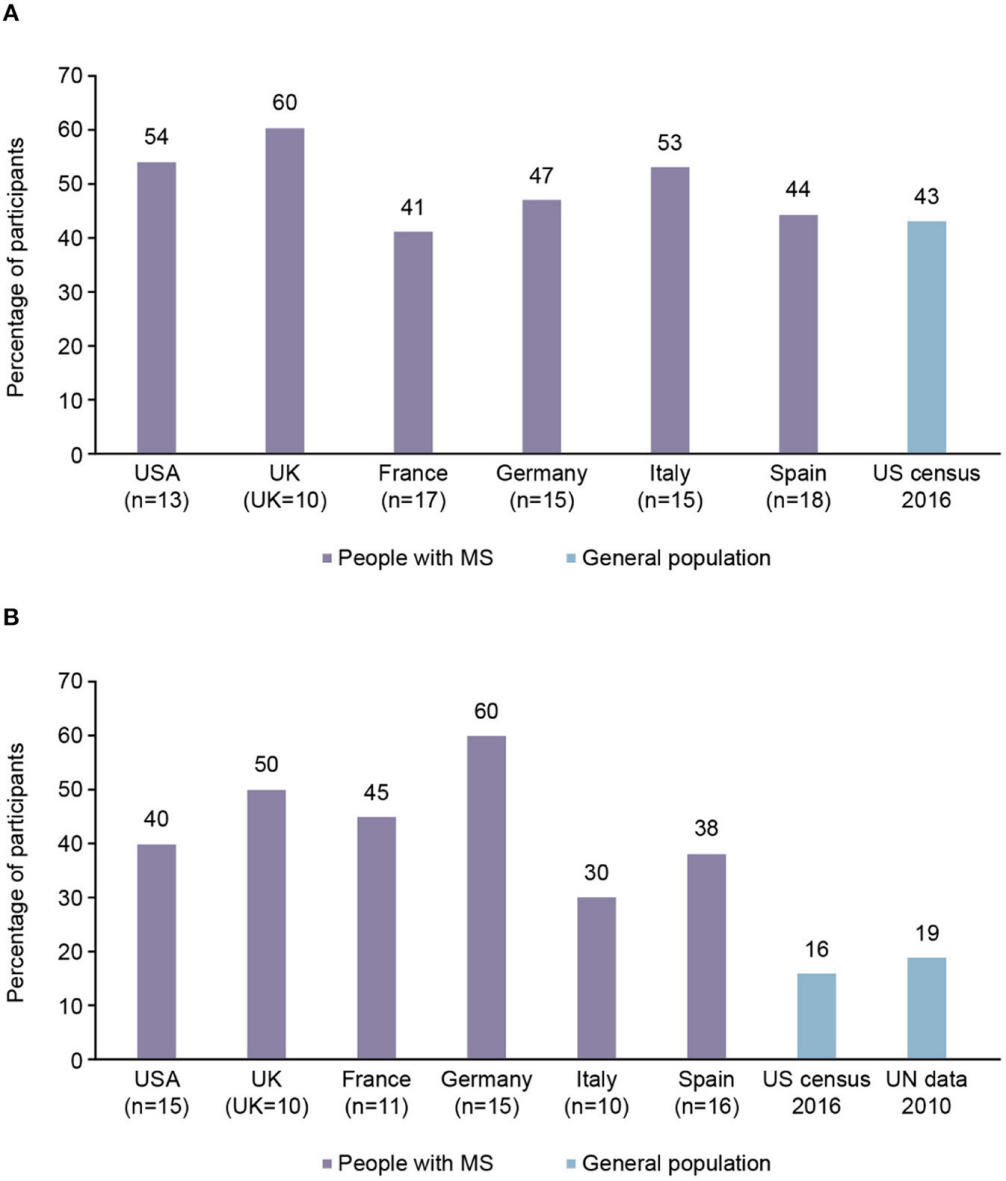
Proportion of participants with multiple sclerosis (MS) from each country aged 26–45 years with no children, compared with general population findings. **(A)** 26–35 years of age subgroup. **(B)** 36–45 years of age subgroup.

On average, 49% of female participants in the 26–35 years of age subgroup had no children compared with 43% of females of the same age surveyed in a 2016 US census (Figure 2A). In the 36–45 years of age subgroup, an average of 44% of the female participants had no children compared with 16–19% of females of the same age in the general population, according to data from US and United Nations censuses (from 2016 to 2010, respectively; Figure 2B). The 18–25 years of age subgroup was not analyzed due to a small subgroup population.

### Impact of MS on Family Planning

**Figure 3:**
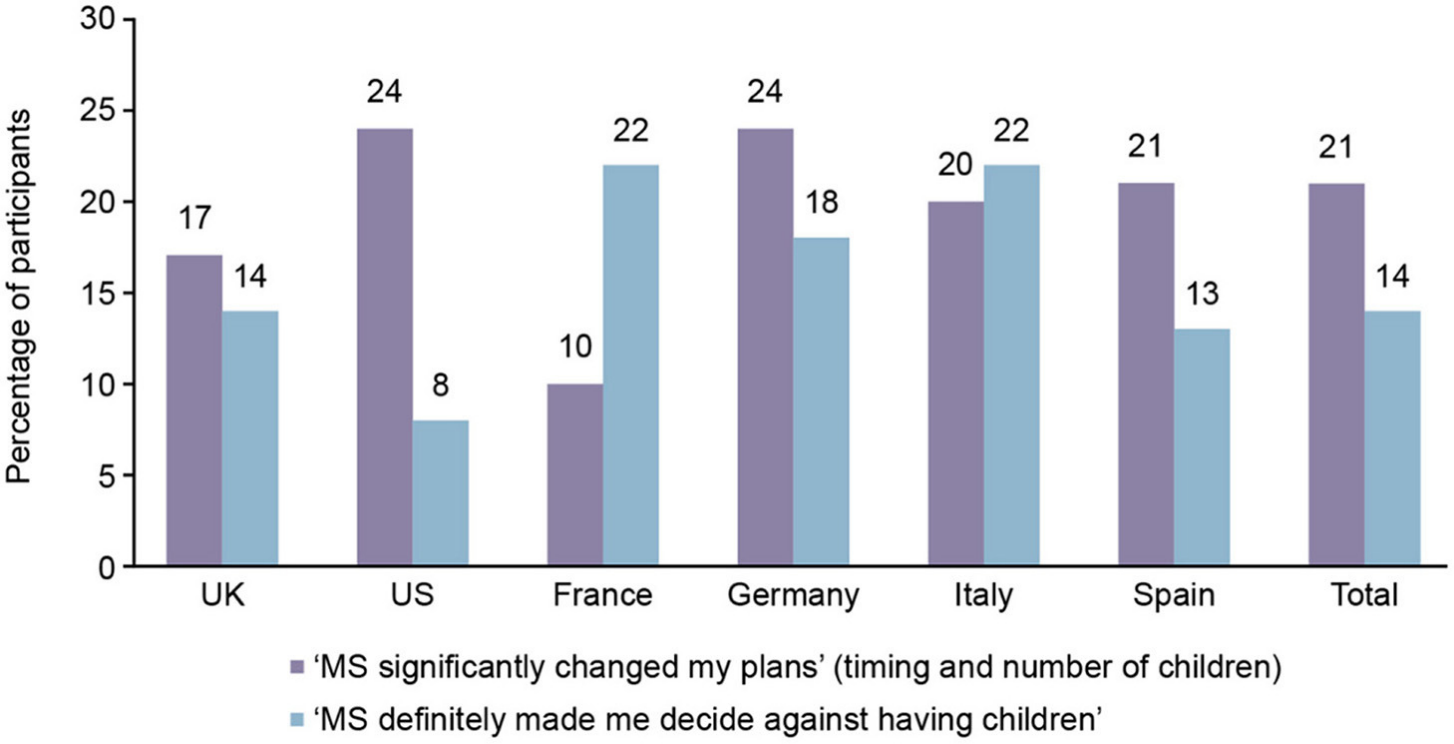
Participants who were significantly impacted by multiple sclerosis (MS) on having children, by country.

Overall, 116/332 (35%) of participants stated that the disease significantly impacted their plans for having children. Of these, 69/116 (59%) significantly changed their timing plans and the number of children they planned to have; 47/116 (41%) decided against having children (Figure 3).

Of the 35% of participants who stated that the disease significantly impacted their plans for having children, the reasons included: the ability to care for the baby while having MS (69%), the risk of the MS condition worsening (47%), the risk of harm to the baby by MS medication (38%), the risk of the baby also having MS (31%), and other reasons (11%).

For the remaining (216/332) participants, 22/216 (10%) indicated the disease delayed their plans for having children, 50/216 (23%) stated that MS led to minimal impact, and 144/216 (67%) indicated no impact.

**Figure 4:**
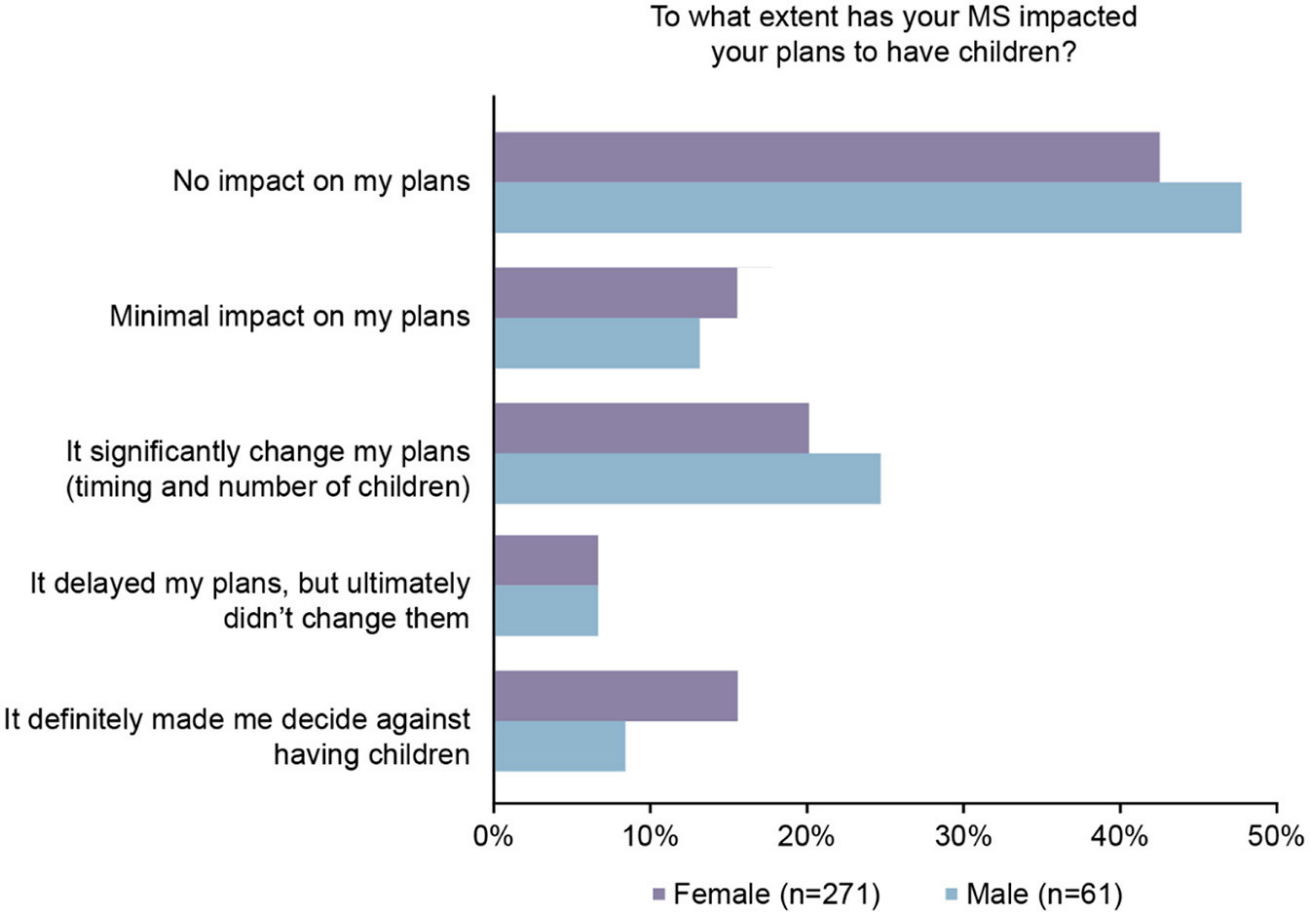
Patients who were significantly impacted by multiple sclerosis (MS) on having children, by gender.

Comparing male and female MS participants' responses, the impact of MS on family planning differed between the genders; men were less likely to say it made them decide against having children than women (Figure 4).

### Sources of Information for Family Planning

**Figure 5:**
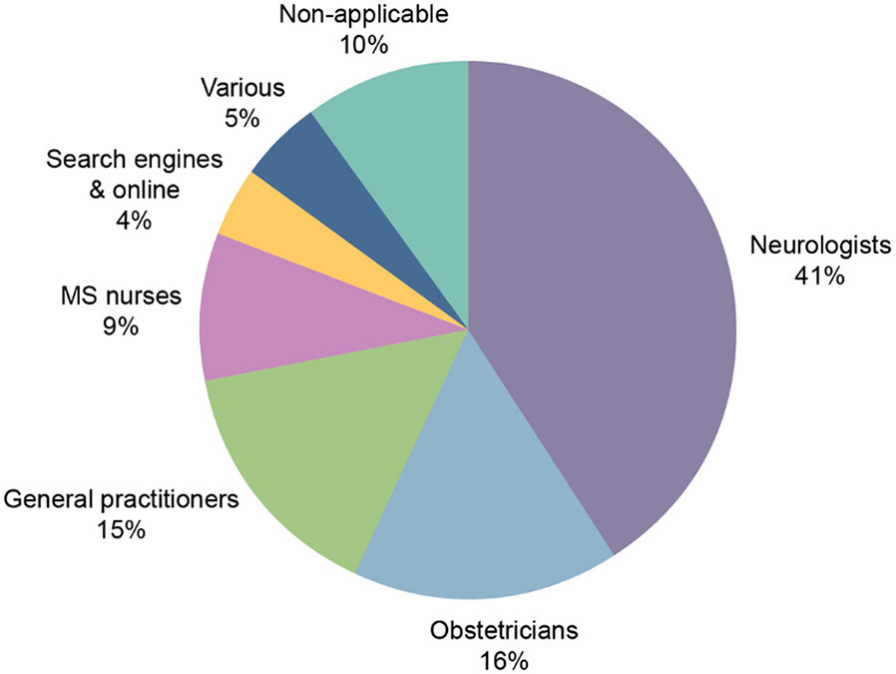
Primary sources of information on family planning. MS, multiple sclerosis.

**Figure 6:**
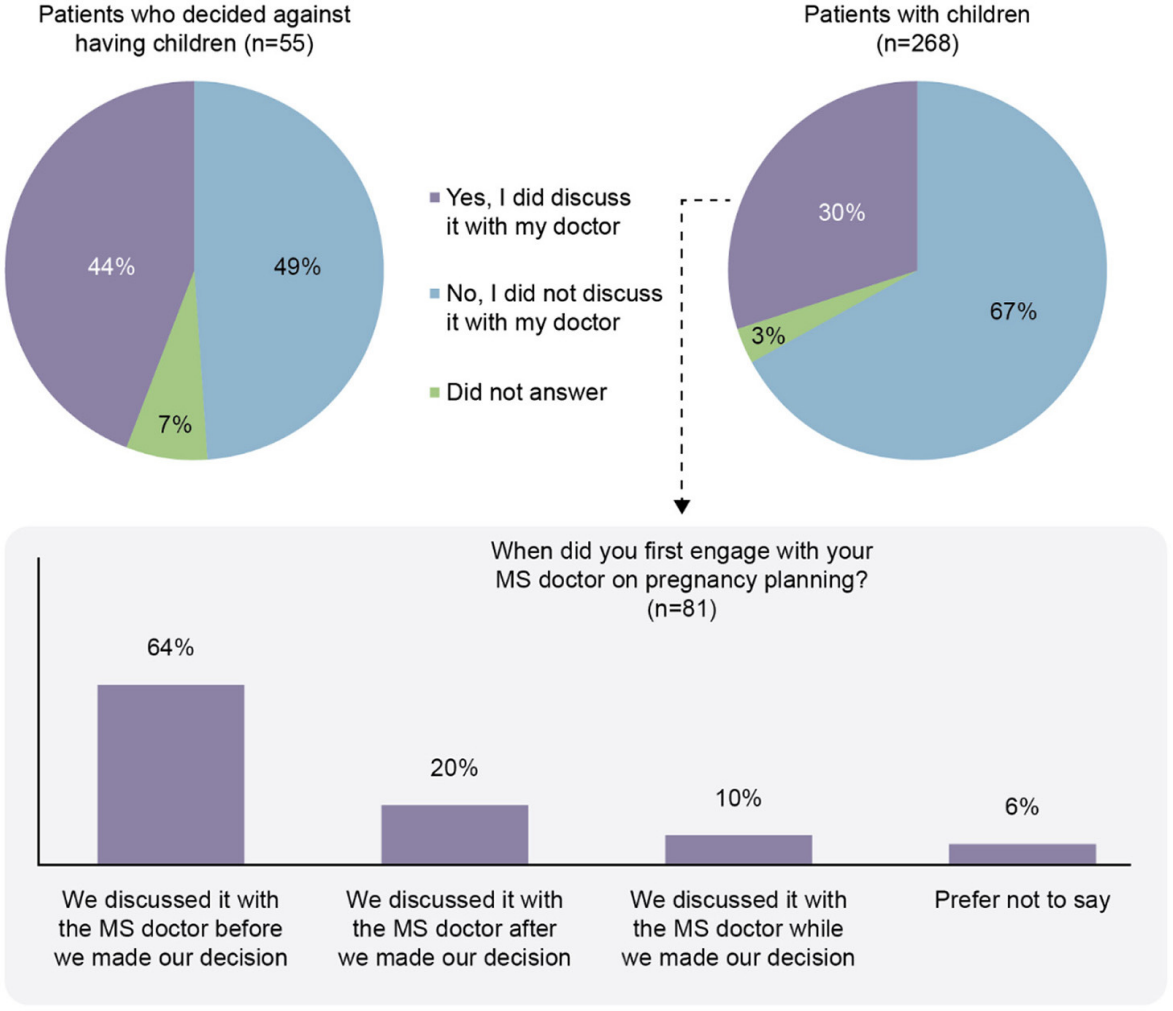
Discussion of family planning with the doctor responsible for management of the participant with multiple sclerosis (MS).

Participants indicated that healthcare professionals (HCPs) were the primary source of information for family planning decisions (81%; Figure 5). Neurologists were found to be the primary source in 41% of responses. Of the patients with children (*n* = 268), only 81/268 (30%) had discussed the topic of having children with their MS physician. The patients who discussed having children with their MS physician tended to discuss pregnancy planning before making a decision (64%; Figure 6).

### Impact of Family Planning on MS Treatment

The timing of planned pregnancy was not considered when selecting treatment by 258/332 (78%) of the participants.

## Discussion

This survey aimed to understand not only family planning decision making in pwMS but also to identify their unmet educational needs in this regard. It was found that pwMS were more likely to have no children than the general population, particularly in the subgroup of patients aged 36–45 years. In a previous online survey, MS diagnosis was found to dramatically impact on wMS in that they were more likely to renounce having children because of the disease, and in pro-parenthood pwMS it impacted especially on having a second child (21). A total of 56% of pwMS reported that the disease affected, with different degrees of impact, their family planning decision making. However, this conflicted with a previous study in Italian pwMS, where only 29% of patients responded that the diagnosis of MS delayed their decision to become a parent (22). A 2018 study of US pregnancy rates found that there were significantly more pregnant wMS in 2014 than in 2006 (23). This may reflect the results obtained from the surveyed pwMS, where those in the 26–35 year age group that had children were comparable to census data, whereas there was a larger disparity for the 36–45 year age group. The accumulation of knowledge about pregnancy outcomes in wMS, as summarized by a number of recent evidence-based reviews on the topic (24–26), may also have impacted the decision making process in recent years.

Among the surveyed participants, the most cited reason for MS to impact family planning was the concern about the ability to care for children whilst affected by MS. The results of the survey are therefore consistent with other previous studies that have investigated the impact of MS on family planning decision making. A 2013 study examining the reproductive practices and attitudes of North Americans diagnosed with MS found that, amongst 5,949 survey participants, the main MS-related reason for not becoming pregnant following MS diagnosis was the perception that symptoms would interfere with parenting (71.2%), followed by concerns of burdening their partner (50.7%) and of the baby inheriting MS (34.7%) (27). The concern that MS symptoms may interfere with raising a child may also be heightened in the period immediately following birth. A meta-analysis from 2011, across 23 MS studies, demonstrated that although relapses per year for pregnant wMS fell from 0.44 in the year before pregnancy to 0.26 during pregnancy, this figure increased to 0.76 relapses per year in the post-partum period (28). However, the impact of treatment on relapses in these patients was not assessed.

Among pwMS who responded that the disease significantly impacted on their family planning decisions, 14% decided against having children entirely. Men were found to be less likely to respond that MS made them decide against having children than women; however, this result is not conclusive as there were significantly fewer men in the survey than women. A similar 2019 study in pwMS in Italy found that only 7% of MS patients decided against having children after being diagnosed with diagnosis (22). Cultural differences between countries may account for the varying responses to the impact of MS on family planning, and a further study to evaluate the impact of cultural differences on family planning would be desirable. However, the real impact of MS on family functioning is yet to be established thus prompting the use of new tools to evaluate the internal and interpersonal family factors (29).

The number of pwMS with children reported in the survey aligns with previous, similar investigations. A 2015 study assessing family planning in wMS in France, for example, found that the mean number of children per woman with MS was 1.37, compared with 1.99 children per woman in the general population in France (30). This reflects the results of our survey, wherein all surveyed countries the number of MS participants within the 36–45 age group with children were reduced in comparison with the general population.

HCPs were determined as the main source of information for family planning for survey participants. Information was primarily received from neurologists with 41% of responses. The approach of neurologists to family planning in MS is rapidly changing; as such, responses given now may not reflect the results obtained from studies several years ago. Our survey found only 4% of participants primarily received family planning information through online search engines and websites. Given the availability of information online, this result was surprising. A potential reason for this may be that resources available to pwMS seeking family planning are specialized and difficult to understand. HCPs may be able to provide the patients with clearer direct information. The Italian MS patient study found similar results, with 39% of patients counseled by a physician to plan pregnancy (22). A 2018 study on family planning in pwMS from Denmark found that 27% of women and 34% of men received family planning information from an internet source. It was also determined that 22 and 41% of female and male pwMS, respectively, received information from their neurologist (31). wMS may feel more comfortable receiving family planning information from an obstetrician or gynecologist compared to other HCPs or information sources due to the specialized nature of their role. Responses from a survey of female neurologists in the USA and Canada in 2004 found that the majority of neurologists refer their patients to an obstetrician-gynecologist to discuss family planning, and many of neurologists were unsure whether their patients used contraception or the type used (32).

The majority of pwMS with children did not discuss the topic of family planning with their doctor. This may be seen as a potential area for improvement, where doctors may need to be more proactive in discussing family planning with their patients. A recent UK consensus recommended that at diagnosis, all wMS of childbearing age should have pre-pregnancy counseling that is repeated at regular intervals, as well as discussions with their MS HCP should they be considering pregnancy (33). Early discussions such as these with a doctor may help alleviate concerns pwMS have regarding decisions on family planning.

Nowadays, family planning influences the selection of DMT as determined in a recent 2018 questionnaire-based study of wMS in Switzerland (17). In this regard, the availability of DMTs with no safety concerns when administered during pregnancy is a valid opportunity to minimize the risk of reactivation of the disease in those wMS attempting to get pregnant.

The limitations of this cross-sectional smartphone-based survey are that the survey also does not allow for deeper reasoning behind responses, consequently leading to generalization issues. Furthermore, the mix of patients may be representative of a self-selected sample and not necessarily of the MS population at large. However, the ease of access to a smartphone-based survey allows participants to be included on a worldwide scale within a short period. The low number of patients surveyed in each country restricts interpretation of findings in comparison to census data, however total patient numbers provide a good indication of family planning trends as a whole. Older patients, such as those in the 45–65 years' bracket, were included in the survey to ensure we had a retrospective view of their family planning decisions. For younger patients, of childbearing potential, those decisions are clearly current; therefore, we focused on findings for this cohort in the paper. Further limitations were that as the study population was not designed to be representative of any specific MS phenotype, there was no data collected about disease characteristics (i.e., disability, disease activity) and there was a lack of a control group within the survey for a direct comparison. The lack of a control group was compensated by using census data from the USA and UN to compare results from survey participants to the general population. Although statistical data by country exists (such as Italian ISTAT data), this form of comparison was not used in the present study. However, census data allowed for a broader population to be used when comparing data groups, reducing selection bias.

In conclusion, MS was found to significantly impact family planning decision making with pwMS significantly less likely to have children in comparison with the general population. Although most survey participants received their family planning information from an HCP, the majority of pwMS with children did not discuss family planning with their doctor. Those that did discuss family planning were most likely to bring up the topic before a decision was made, suggesting that HCPs have a large bearing on family planning decision making for pwMS.

## Data Availability Statement

The raw data supporting the conclusions of this article will be made available by the authors, without undue reservation.

## Ethics Statement

Ethical review and approval was not required for the study on human participants in accordance with the local legislation and institutional requirements. The patients/participants provided their written informed consent to participate in this study.

## Author Contributions

SB and LL: contributed to the analysis of the results and the writing of the manuscript. HW: responsible for study design, study management, and reporting. SR and DJ: contributed to the interpretation of the results and the writing of the manuscript. All authors contributed to the article and approved the submitted version.

## Conflict of Interest

SB received speaker honoraria and/or travel grants and/or Advisory board fees from Teva, Merck-Serono, Sanofi-Genzyme, Novartis, Biogen, and Roche. LL received speaker honoraria and travel grants from Teva, Merck KGaA (Darmstadt, Germany), Sanofi, Novartis, Biogen, Roche, and Bayer. HW is an employee of Aequus Research. SR is an employee of EMD Serono Research & Development Institute Inc., a business of Merck KGaA, Darmstadt, Germany. DJ is an employee of Merck KGaA, Darmstadt, Germany. The authors declare that this study received funding from EMD Serono Research & Development Institute, Inc., Billerica, MA, USA; a business of Merck KGaA, Darmstadt, Germany. The funder had the following involvement with the study: data interpretation and manuscript preparation. Medical writing assistance was provided by Joseph Ward of inScience Communications, Springer Healthcare Ltd., Chester, UK, and was funded by Merck KGaA, Darmstadt, Germany.
