# Supplementary material for: Family Planning Decision Making in People With Multiple Sclerosis
Source: Front Neurol. 2021 Apr 28;12:620772. doi: 10.3389/fneur.2021.620772 (PMC8113643; doi:10.3389/fneur.2021.620772)
Supplement: Supplementary file 1 [file Data_Sheet_1.PDF]

Supplementary Material

Survey Questions

- When did you start taking your current medication?
- How many children do you have?
- Are you planning to have children/more children in the next 2-3 years?
    - Yes / No
- To what extent has your MS impacted your plans of having children?
    - No impact on my plans
    - Minimal impact on my plans
    - It delayed my plans, but ultimately didn't change them
    - It significantly changed my plans (timing & number of children)
    - It definitely made me decide against having children
- What sources of information have you consulted for family planning in relation to your MS?
    - Neurologist - How important was this source for you?
        - Very important
        - Fairly important
        - Neutral
        - Fairly unimportant
        - Not at all important
    - OB/GYN - How important was this source for you?
    - MS Nurse - How important was this source for you?
    - GP/FP/PCP - How important was this source for you?
    - Online source - please specify - How important was this source for you?
    - Other - please specify - How important was this source for you?
    - None of the above
- Was family planning a consideration when selecting your MS treatment?
    - Yes / No
- What sources of information have you consulted for your MS drug choice?
    - Neurologist - How important was this source for you?
    - OB/GYN - How important was this source for you?

- MS Nurse - How important was this source for you?
- GP/FP/PCP - How important was this source for you?
- Online source - please specify - How important was this source for you?
- Other - please specify - How important was this source for you?
- None of the above
